# Comprehensive GC-MS Profiling and Multi-Modal Pharmacological Evaluations of *Haloxylon griffithii*: In Vitro and In Vivo Approaches

**DOI:** 10.3390/ph18060770

**Published:** 2025-05-22

**Authors:** Iram Iqbal, Mohamed A. M. Ali, Fatima Saqib, Kinza Alamgir, Mohammad S. Mubarak, Anis Ahmad Chaudhary, Mohamed El-Shazly, Heba A. S. El-Nashar

**Affiliations:** 1 Department of Pharmacology, Faculty of Pharmacy, Bahauddin Zakariya University, Multan 60800, Pakistan; iramiqbal.bzu@gmail.com; 2 Health & Population Department, Government of the Punjab, Lahore 54000, Pakistan; 3 Department of Biology, College of Science, Imam Mohammad Ibn Saud Islamic University (IMSIU), Riyadh 11623, Saudi Arabia; 4 Department of Bioinformatics and Biotechnology, Government College University Faisalabad (GCUF), Faisalabad 38000, Pakistan; kinzaalamgir99@gmail.com; 5 Department of Chemistry, The University of Jordan, Amman 11942, Jordan; 6 Department of Pharmacognosy, Faculty of Pharmacy, Ain Shams University, Abbassia, Cairo 11566, Egypt

**Keywords:** *Haloxylon griffithii*, spasmolytic, ADME, GCMS, calcium channel blocker, antidiarrheal

## Abstract

**Background/Objectives:** *Haloxylon griffithii* is a medicinal plant possessing therapeutic effects in disorders associated with the gastrointestinal (GIT) system. This research aims to study the pharmacological activity of *Haloxylon griffithii* in a multidimensional manner, involving phytochemistry screening and in vitro and in vivo experiments. **Methods:** The whole dried plant was extracted with 80% methanol and further fractionation using solvents of increasing polarity. GC-MS analysis was performed on the crude extract to discover volatile compounds. The spasmolytic/spasmogenic effect was assessed in isolated rabbit jejunum using spontaneous and K⁺-induced contractions, as well as contractions induced by increasing concentrations of calcium ions in depolarized tissue. Antidiarrheal activity was evaluated in Swiss albino rats/mice (*n* = 6/group) using castor oil-induced diarrhea and peristaltic index models. In silico ADMET screening was conducted via SwissADME and pkCSM. **Results**: The GC-MS profiling of *H. griffithii* revealed the presence of 59 phytochemicals and a rare azulene derivative and constituents, including α-santonin and hexadecanoic acid esters, with favorable pharmacokinetic profiles, as predicted using SwissADME and pkCSM computational tools. The in vitro and in vivo experiments revealed the significant calcium channel blocking activity in non-polar fractions (*n*-hexane and ethyl acetate), while the polar extracts (ethanolic, aqueous) exhibited cholinergic effects, indicating a dual mode of action. **Conclusions**: This was a first-time demonstration of both antidiarrheal and smooth muscle-relaxant activity in *H. griffithii*, supported by GC-MS profiling and pharmacological assay. The findings lend scientific credibility to the traditional use of the plant in community healthcare, while also reinforcing the need for further pharmacological and clinical studies to explore its potential in drug development.

## 1. Introduction

Natural products have long been a cornerstone in traditional healing practices and modern therapeutic approaches. Scientists today are increasingly focused on understanding how plant-based medicines exert their effects at the molecular level. Nature remains a rich source of powerful and diverse chemical compounds, offering immense value to drug discovery. However, defining the specific actions of these substances remains difficult, mainly due to their complex activity and tendency to influence multiple biological targets [1].

Recent technological advancements have greatly facilitated the study and utilization of medicinal plants [2,3,4,5]. These methods enable rapid screening and modeling of functional compounds, accelerating the identification of potential therapeutic agents by predicting interactions between plant-derived compounds and biological targets [6,7].

Pakistan’s diverse and hospitable climate makes it a rich reservoir of medicinal plants, positioning the country as a major player in the global herbal and pharmaceutical industries. Out of the 6000 known medicinal plant species worldwide, Pakistan is home to approximately 400 to 600, highlighting its significant role in offering natural remedies for various health conditions [8]. *Haloxylon griffithii* has drawn particular attention for its pharmacological potential among Pakistan’s medicinal flora. Predominantly found in Northern Baluchistan, this plant is rich in secondary metabolites, contributing to its antioxidant, antimicrobial, and anti-inflammatory properties [9,10]. Its bioactive compounds, including flavonoids, alkaloids, and terpenoids, are crucial for developing therapeutic agents while enhancing plant resilience against pathogens and environmental stressors. Traditionally, *H. griffithii* has been used to treat gastrointestinal disorders, with studies supporting its role in modulating gut microbiota and reducing gastrointestinal motility [9,10]. Additionally, published research has demonstrated its antimicrobial activity against pathogenic bacteria like *Escherichia coli* and *Staphylococcus aureus*, both responsible for gastrointestinal infections. Additionally, phenolic constituents enhance its antioxidant potential, further validating its use in inhibiting oxidative stress and inflammation in the GIT. Scientific evidence also indicates that different extracts of *H. griffithii* possess anti-urease action, which is beneficial for controlling ailments such as peptic ulcers caused by *Helicobacter pylori* infection [11].

Despite the widespread use of medicinal plants, a significant gap remains in the scientific evaluation of their biological and pharmacological properties [8,12,13]. It is estimated that fewer than 20% of medicinal plants have been rigorously studied, highlighting an urgent need for comprehensive research [5,12,13,14,15]. Validating the efficacy and safety of these plants through pre-clinical and clinical studies is essential for integrating traditional remedies into modern medicine [16,17]. Expanding research efforts will allow underexplored plants like *Haloxylon griffithii* to be fully utilized, paving the way for novel drug discoveries and improved global health outcomes. Based on the previous discussion, the present work aimed to identify *H. griffith*’s medicinal relevance and how it can be applied in pharmaceutical formulations. As little information is available about this plant, our research will focus on screening it for its effect on the GIT and the mechanisms involved.

## 2. Results

### 2.1. GC-MS Analysis

**Figure 1:**
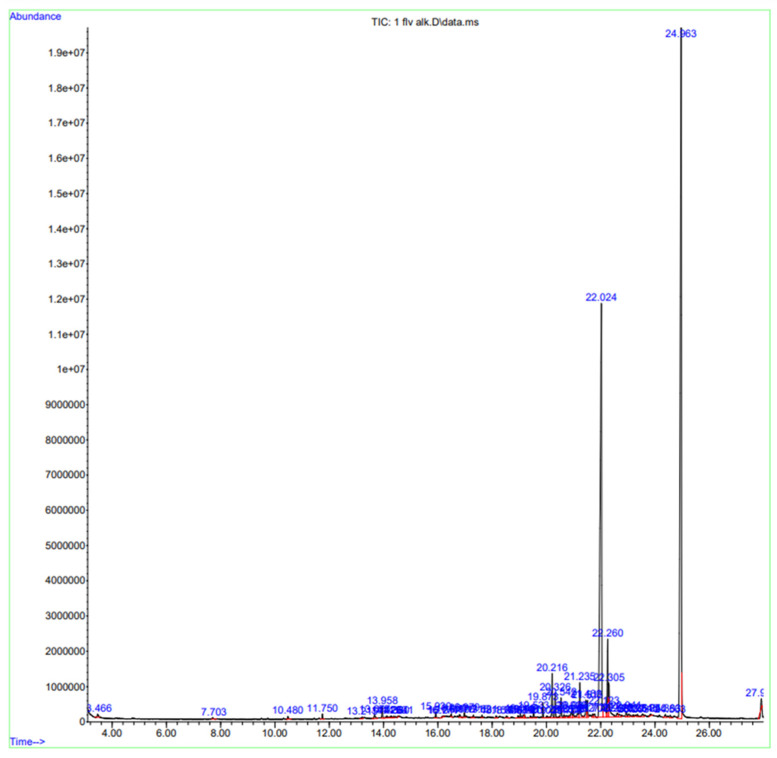
GC-MS chromatogram of volatile oil extracted from *Haloxylon griffithii.*

The prime phytochemical components of the hydroethanolic extract were evaluated using GC-MS. The chromatogram shows the presence of 59 compounds in a given run time (Figure 1). Based on a NIST.20L and DEMO.L database comparative analysis of the compounds with a hit quality above 60, 23 components, listed in Table 1 and Table A2, were identified as major components. The complete list of all 59 identified compounds, including their retention times, peak areas, and spectral match scores, is provided in Table A1 for transparency and reference. Several compounds identified in this study have previously been unreported in *H. griffithii*, representing a novel contribution to its phytochemical profile. Notably, the detection of *o*-cymene and 2,4-di-*tert*-butylphenol, a potent antioxidant commonly found in other medicinal plants [6], represents a novel addition to the phytochemical repertoire *of H. griffithii*. Furthermore, the identification of a rare azulene derivative, ((3aR,4R,7R)-1,4,9,9-tetramethyl-3,4,5,6,7,8-hexahydro-2H-3a,7-methanoazulen-2-one) was also worth mentioning. The presence of complex aromatic hydrocarbons such as benzene, 1,2,4,5-tetrakis(1-methylethyl)- and benzene, hexaethyl- further emphasizes the chemical richness of the extract. This is the first documentation of these specific compounds in *H. griffithii*, underscoring the plant’s untapped potential and supporting its ethnomedicinal use in gastrointestinal disorders.

### 2.2. In Silico Models

#### ADME Profiling of GC-MS-Identified Compounds of *H*. *griffithii*

The in silico ADMET assessment of the phytochemicals derived from a GC-MS analysis of *Haloxylon griffithii* revealed a diverse array of pharmacokinetic and toxicity characteristics. Only compounds that satisfied essential criteria such as good GIT absorption, non-inhibition of major metabolic enzymes, and compliance with drug-likeness rules (e.g., Lipinski, Veber) were retained for detailed profiling (Table 2). Some compounds like hexadecanoic acid ethyl ester, its methyl counterpart, and *n*-hexadecanoic acid showed strong GIT absorption, lacked P-glycoprotein interactions, and favorable clearance parameters, highlighting their potential for effective oral delivery. In contrast, more lipophilic substances, such as (E)-9-octadecenoic acid ethyl ester and bis(2-ethylhexyl) phthalate, were obvious from the elevated total clearance rates (log mL/min/kg > 1.8). However, some were limited by poor GI absorption and multiple violations across the drug-likeness filters like Lipinski and Veber. In this context, α-santonin and o-cymene appeared as the most favorable in terms of drug-likeness, meeting all criteria set by Lipinski, Veber, and Egan rules and exhibiting favorable blood–brain barrier permeability, thus suggesting possible CNS-targeted activity. Meanwhile, benzene and hexamethyl combined good lipophilicity and broad tissue distribution but labeled potential hepatotoxicity, demanding more in-depth toxicological profiling. Positively, many of these compounds did not interfere with key CYP450 enzymes, indicating a low risk of metabolic interactions.

### 2.3. In Vitro Experiments

#### Effects of *H. griffithii* Extracts on Isolated Tissues of Rabbit Jejunum

**Figure 2:**
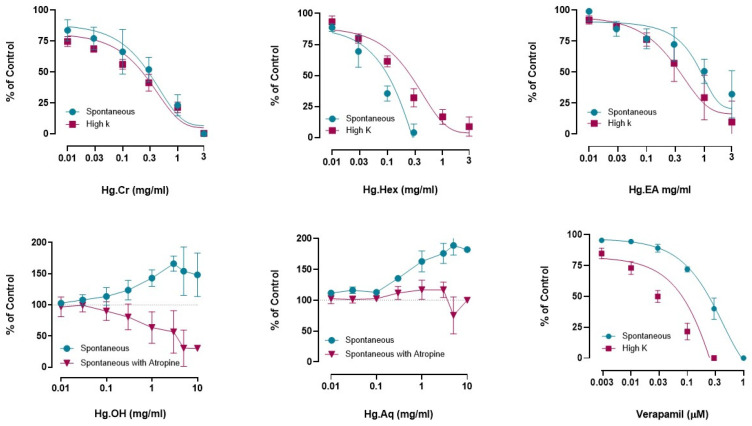
Effect of Hg.Cr, Hg.Hex, Hg.EA, and verapamil on jejunal spontaneous and K^+^ (80 mM)- induced spastic contractions Hg.OH and HG.Aq on spontaneous and atropine-treated contractions. (Data expressed as the mean ± SEM, *n* = 5; data were analyzed by sigmoidal dose–response curve).

The effects of crude (Hg.Cr), *n*-hexane (Hg.Hex), ethyl acetate (Hg.EA), ethanol (Hg.OH), and aqueous (Hg.Aq) extracts of *H. griffithii* were tested in spontaneously contracting jejunum preparations. Hg.Cr, Hg.Hex, and Hg.EA showed spasmolytic action by inhibiting the spontaneous rhythmic contraction in a dose-dependent way, with EC_50_ values of 0.65 mg/mL (0.29 to 1.61; 95% CI), 0.094 mg/mL (0.040 to 0.25; 95% CI), and 1.83 mg/mL (0.52 to 18.1; 95% CI), respectively. The results are comparable to verapamil, which has a spasmolytic action with an EC_50_ value of 0.43 mg/mL (0.30 to 0.65; 95% CI). On the contrary, when exposed to rhythmic or periodic jejunal contractions, Hg.OH and Hg.Aq exhibited dose-dependent contractility (Figure 2). The spasmogenic effects of Hg.OH and Hg.Aq were diminished, and a mild dose-dependent spasmolytic effect was seen in an atropine (1 µM)-pretreated jejunum preparation. In addition, the Hg.Cr. extract exhibited a dose-dependent inhibition to the sustained contractions of K^+^ (80 mM), with an EC50 of 0.47 mg/mL (0.30 to 0.77; 95% CI). Hg.Hex also inhibited the K^+^ (80 mM)-induced contraction in a dose-dependent manner, with an EC_50_ of 0.14 mg/mL (0.091 to 0.20; 95% CI), and Hg.EA also caused inhibition of K^+^ (80 mM)-induced contraction, with an EC50 of 0.41 mg/mL (0.18 to 0.96; 95% CI). These results are comparable to verapamil, which caused relaxation of K^+^ (80 mM)-induced contraction, with an EC_50_ of 0.038 mg/mL (0.024 to 0.060; 95% CI). Both Hg.OH and Hg.Aq did not cause relaxation in high K-induced contractions (Figure 2).

**Figure 3:**
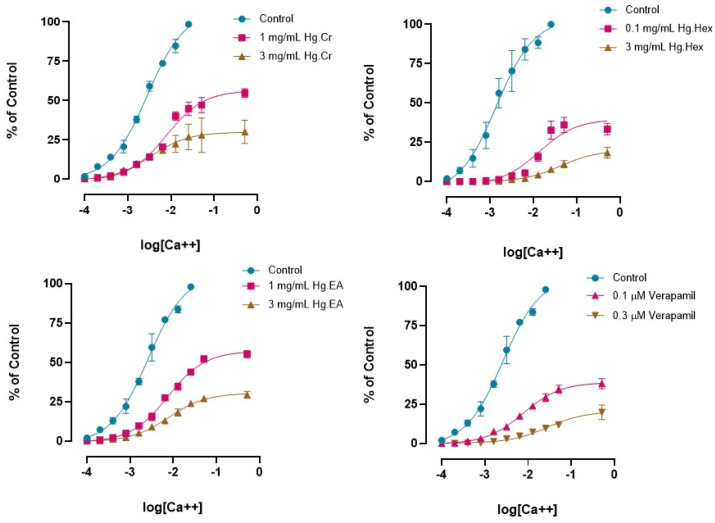
Effect of Hg.Cr, Hg.Hex, Hg.EA, and verapamil on concentration response curves of calcium (CRCs) (data are expressed as the mean ± SEM, *n* = 5; data were analyzed by sigmoidal dose–response curve).

Concentration response curves (CRCs) of calcium in the absence and presence of extract were constructed to further confirm the calcium channel antagonism response of the extracts Hg.Cr, Hg.Hex, and Hg.EA. All these extracts caused the rightward shifting with suppression of calcium CRCs at 0.1 and 3 mg/mL (Figure 3).

### 2.4. In Vivo Experiments

#### 2.4.1. Antidiarrheal Activity

**Figure 4:**
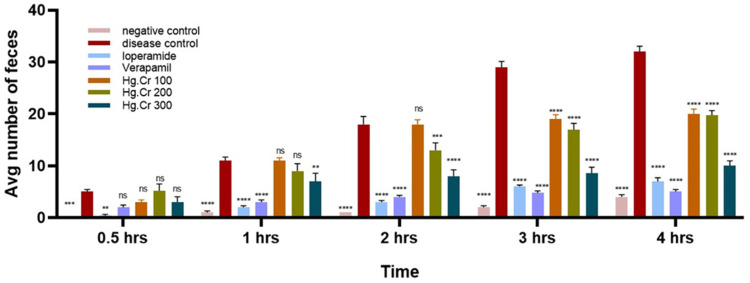
Hg.Cr and loperamide impact on diarrhea brought on by castor oil. Data are expressed as the mean ± SEM (*n* = 5). For analysis, two-way ANOVA with multiple comparison testing was used. While **** *p* < 0.0001, *** *p* < 0.001, ** *p* < 0.01, ns = non-significant in comparison with positive control.

**Figure 5:**
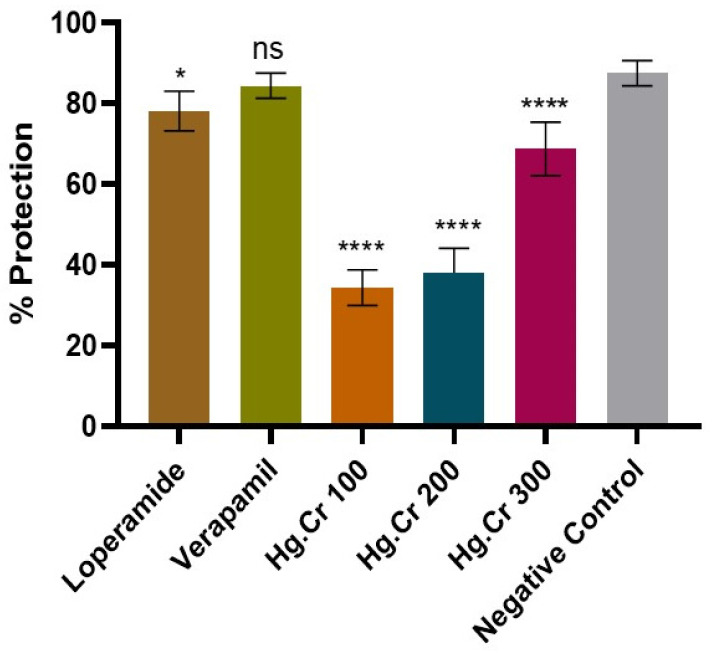
The effect of Dr.Cr on caster oil-induced diarrhea. Data are expressed as the mean ± SEM (*n* = 5). The standard one-way ANOVA testing with multiple comparison tests was used for analysis. While **** *p* < 0.0001, * *p* < 0.05, ns = non-significant compared with negative control.

Results from this investigation showed that Hg.Cr causes a significant decrease in the number of wet feces drops in mice (Table 3, Figure 4). At higher doses, Hg.Cr extract in mice caused significant anti-diarrheal effects compared to the disease control group. Hg.Cr 100 mg/kg, 200 mg/kg, and 400 mg/kg showed 34.4, 38.1, and 68.7% protection, respectively (Table 4, Figure 5).

#### 2.4.2. Effect of *H. griffithii* on Charcoal Meal GIT Transit Time

**Figure 6:**
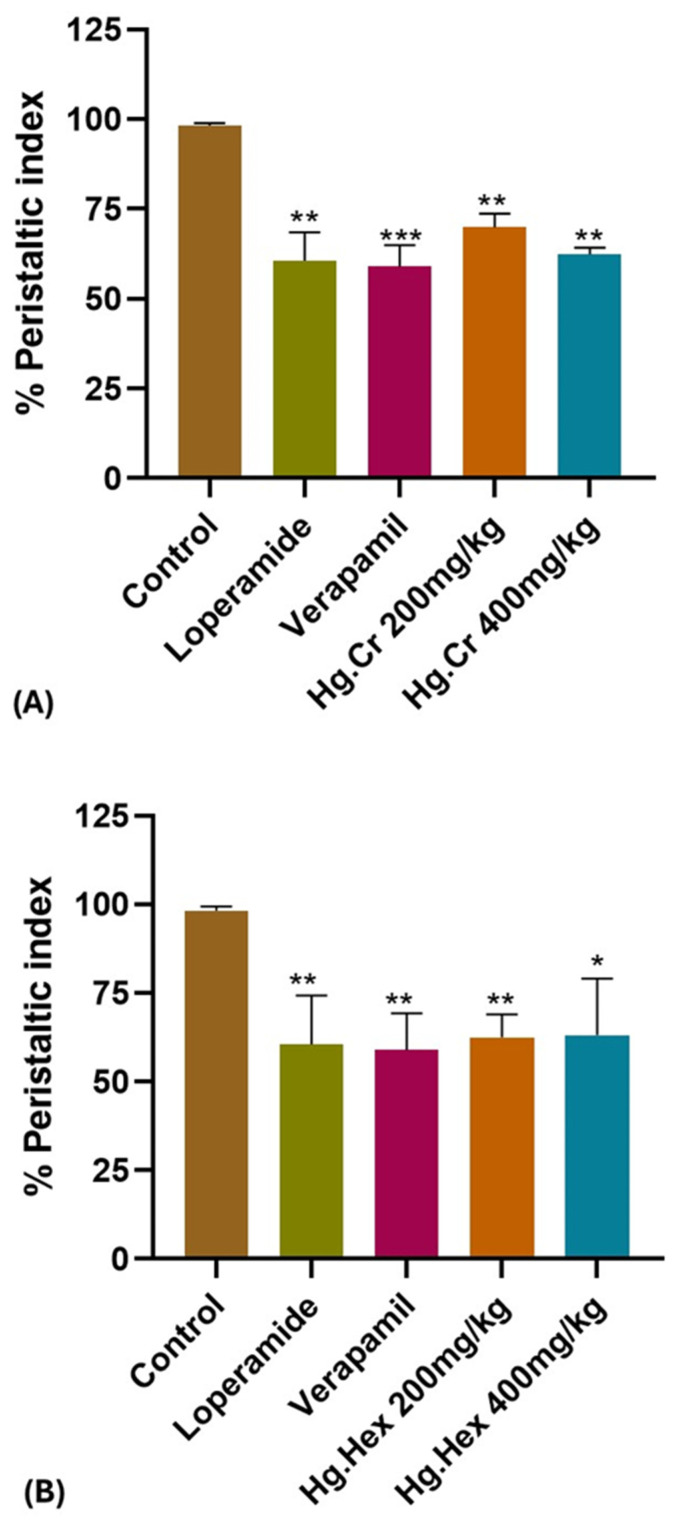

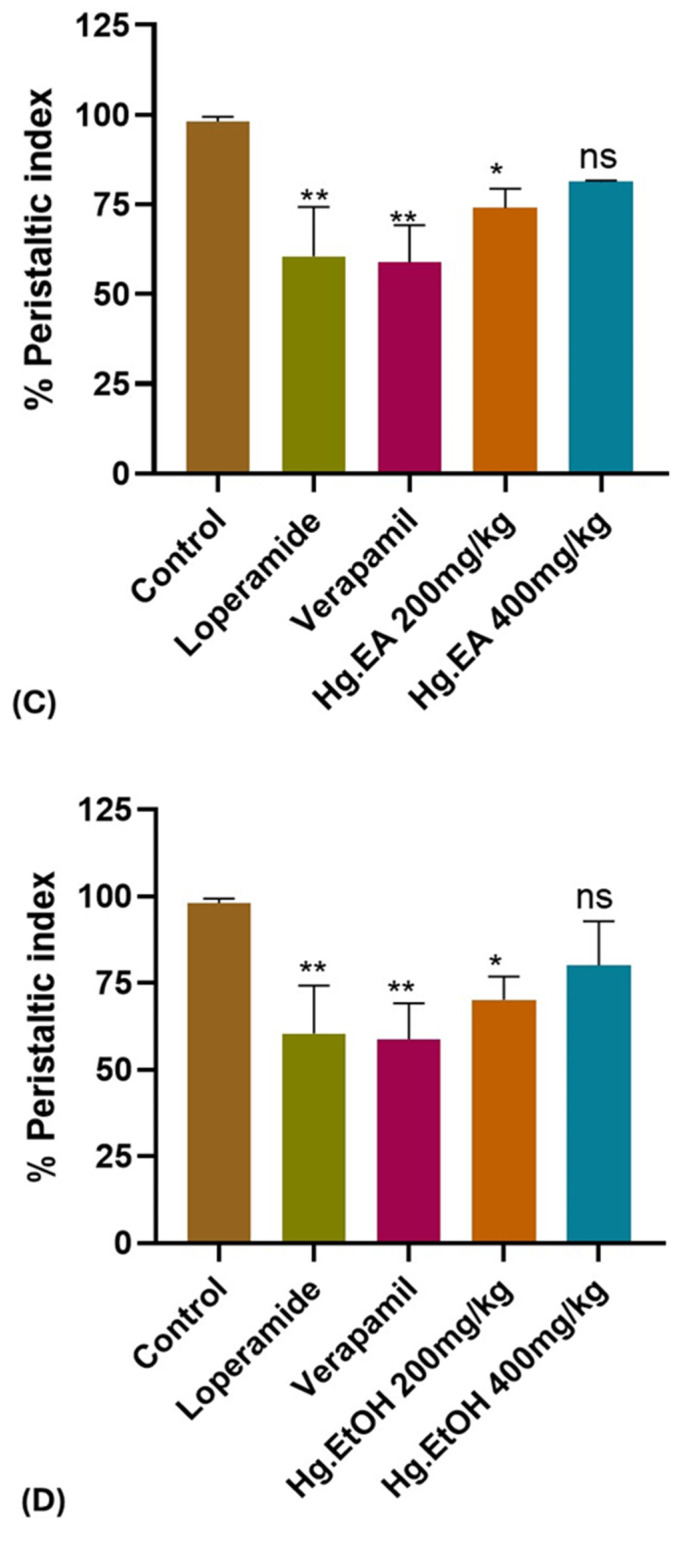

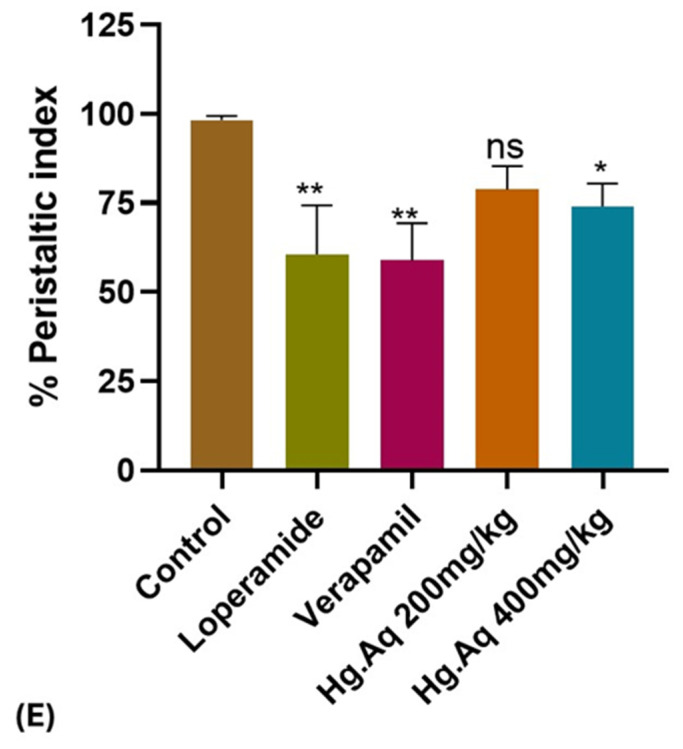
Effect of *Haloxylon griffithii* Extracts (**A**). Hg.Cr (**B**) Hg.Hex, (**C**) Hg.EA, (**D**) Hg.OH, (**E**) Hg.Aq on gastrointestinal motility in comparison to standard antidiarrheal agents. Values are expressed as the mean ± SEM (*n* = 3) *: *p* < 0.05, **: *p* < 0.01, ***: *p* < 0.001, ns: Not significant.

The control group establishes the baseline with a 100% peristaltic index, representing normal GI motility. Loperamide caused a significant drop (*p* < 0.01) in the peristaltic index. Verapamil also significantly (*p* < 0.01) reduced the peristaltic index compared to the control. Hg.Cr doses of 200 and 400 mg/kg significantly (*p* < 0.01 to *p* < 0.001) reduced the peristaltic index, suggesting significant activity of the crude extract. In contrast, Hg.Hex caused significant drops (*p* < 0.05 to *p* < 0.01), especially at the higher dose (400 mg/kg). This suggests potent activity due to nonpolar bioactive compounds in the *n*-hexane fraction. In the Hg.EA, significant effects were noted at 200 mg/kg (*p* < 0.01). However, no significant reduction was observed at 400 mg/kg. This may indicate a plateau effect or reduced bioavailability at higher doses. On the other hand, Hg.EtOH caused significant reductions at 200 mg/kg (*p* < 0.01) but became nonsignificant (NS) at 400 mg/kg, similar to Hg.EA. Finally, Hg.Aq showed no significant effect at 200 mg/kg, while a mild reduction (*p* < 0.05) was observed at 400 mg/kg (Figure 6).

## 3. Discussion

The genus Haloxylon was traditionally used as medicine among local healers [9]. Many plant-derived compounds are potential medicinal agents based on their pharmacokinetic properties. These compounds can be screened through ADMET analysis [18]. *Haloxylon griffithii* extract contains several bioactive phytochemicals, identified through GC-MS analysis (Table 1). Numerous investigations revealed that the action of medicinal herbs is predominantly due to their Ca++ antagonistic action, and multiple pathologies can be treated through specific interactions between bioactive molecules and their respective target proteins [19,20].

To validate our hypothesis regarding the antidiarrheal potential of *H. griffithii* via calcium channel blockade, we conducted a broad series of both in vitro and in vivo experiments. The GIT motor tone is regulated by various physiological mediators responsible for the gut’s stimulatory action. Among them, an increase in the cytosolic calcium levels, either by the extracellular influx of calcium or the release of stored calcium in the cytosol of the endoplasmic reticulum, induces membrane depolarization and spontaneous contractions of the Jejunum preparation. Any substance interrupting this process is a confirmed antispasmodic [21,22] and therapeutically valuable for managing hyperactive gastrointestinal conditions [19]. Interestingly, in our in vitro experiments, the results for polar (ethanolic and aqueous) and non-polar (*n*-hexane and ethyl acetate) extracts of *H. griffithii* were opposite.

The crude *H. griffithii* extract and non-polar extracts (*n*-hexane and ethyl acetate) showed significant spasmolytic activity, supporting calcium antagonism, as evidenced by inhibition of high-K⁺-induced jejunal contractions (Figure 2) and a rightward shift in calcium CRCs (Figure 3). Findings showed that high doses of K+ activate voltage-dependent Ca++ channels, causing a flooding of free Ca++ in the cytosol and a profound depolarization of the action potential of the membrane, causing a protracted contraction of a long duration [23]. Reversal of this depolarization causes the relaxation of smooth muscles [24], and substances that inhibit smooth muscle contractions caused by K^+^ (80 mM) levels appear to impede Ca++ influx into the cytosol [23]. In this context, previous research has established that all Ca++ channel blockers exhibit the characteristic of blocking calcium’s slow entry, which can be reversed when Ca++ is introduced [25]. In our study, pretreatment with the extracts followed by the formation of calcium CRCs led to inhibition at doses of 1 and 3 mg/mL, producing a rightward parallel shift in the jejunum tissue preparation, like the effect of verapamil at doses of 0.1 and 0.3 μM [19] (Figure 3). These effects were twinned with those of verapamil, a standard L-type calcium channel blocker. In contrast, polar extracts (ethanolic and aqueous) produced dose-dependent spasmogenic effects. Interestingly, the spasmogenic properties of both ethanolic and aqueous extracts were blocked entirely by atropine (1 µM), pointing to cholinergic compounds likely acting through muscarinic receptor pathways (Figure 2) [26]. This duality highlights the presence of both excitatory and inhibitory phytoconstituents within *H. griffithii*—a notable strength of the study, offering a mechanistic explanation for its traditional use in both diarrheal and cramping conditions.

In an in vivo antidiarrheal model, the extract reduced the frequency and severity of diarrhea, delayed its onset, and exhibited a dose-dependent inhibition of wet stool production at 100, 200, and 400 mg/kg doses compared to the saline-treated group, where protection was absent (Figure 4). Castor oil, upon hydrolysis, forms ricinoleic acid, altering the transportation of electrolytes and water. This interruption leads to contractions in the gastrointestinal tract [27]. Studies show that a potential anti-diarrheal candidate may show its potential by inhibiting bowel contraction by acting on μ-opioid receptors [28] or reducing smooth-muscle contraction [29]. The antidiarrheal effect of the *H. griffithii* extract can be credited to multiple components present in the plant, which have an inhibitory action. In this regard, bioactive compounds such as flavonoids, alkaloids, and saponins inhibit the secretion and motility induced by ricinoleic acid [30,31].

The peristaltic index, used to quantify gut motility [32], further validated these effects. Non-polar fractions significantly reduced intestinal transit, similar to loperamide and verapamil. The crude extract (Hg.Cr) exhibited the most significant decrease in peristaltic index, indicating a synergistic effect of multiple phytochemicals (Figure 6A). Among all fractions, hexane extract (Hg.Hex) demonstrated notable activity (Figure 6B), possibly due to nonpolar compounds substantially contributing to the antidiarrheal effect. The ethyl acetate (Hg.EA) and ethanolic (Hg.EtOH) extracts demonstrated significant reductions in GI motility at lower doses. However, at higher concentrations, their reduced efficacy (Figure 6C,D) suggests possible complex pharmacokinetic interactions requiring further investigation. In contrast, the aqueous extract (Hg.Aq) demonstrated the least activity, indicating that water-soluble compounds in *Haloxylon griffithii* may be less prominent in influencing gastrointestinal motility. These results are correlated with in vitro studies (Figure 2), where atropine-pretreated jejunal tissues did not produce any relaxation after administration of Hg.Aq. The observed reductions in the peristaltic index across various *Haloxylon griffithii* extracts suggest potential antidiarrheal properties, likely due to bioactive compounds such as alkaloids, flavonoids, and tannins. Phytochemical analyses have confirmed the presence of these constituents in *H. griffithii*, supporting their role in modulating gastrointestinal motility [33].

These findings agree with studies highlighting the pharmacological potential of the Haloxylon genus, which includes species traditionally used to treat gastrointestinal disorders [34]. Our findings align with previous research on other medicinal plants whose bioactivity is associated with fatty acid esters, azulene derivatives, and monoterpenes, such as those identified in our GC-MS analysis. For instance, α-santonin and linoleic acid esters have previously been reported to exert antispasmodic and anti-inflammatory actions [35], consistent with our data. Hexadecanoic acid methyl ester has been previously reported for its anti-inflammatory, antioxidant, and antimicrobial activities [36]. Similarly, *p*-cymene has been reported to exhibit gastroprotective effects by enhancing gastric mucus production and reducing oxidative stress [37,38]. It has also demonstrated mild spasmolytic effects, likely mediated through calcium channel modulation and smooth muscle relaxation, supporting its traditional use in relieving intestinal cramps, colic, and spasms [39]. The identification of a rare azulene derivative, ((3aR,4R,7R)-1,4,9,9-tetramethyl-3,4,5,6,7,8-hexahydro-2H-3a,7-methanoazulen-2-one), is new for this species. Azulene derivatives, a structural class to which this compound belongs, are well-known for their spasmolytic and anti-inflammatory properties, often utilized in gastrointestinal and dermatological formulations. This aligns with the findings in pharmacological research on azulenes and related sesquiterpenes, such as bakkenolide and guaiazulene, which exhibit similar bioactivities [40], suggesting potential relevance to the plant’s traditional gastrointestinal applications.

One of the strengths of this study is the integration of multiple in vitro and in vivo experimental models: isolated jejunum relaxation, calcium concentration–response curves, castor oil-induced diarrhea, and peristaltic index assessment. These models collectively confirmed the spasmolytic and antidiarrheal actions of specific extract fractions. Nonetheless, our study has certain limitations. GC-MS profiling is confined to detecting volatile compounds, so it may have missed critical non-volatile phytochemicals, such as alkaloids or glycosides. The bioactivity results are likely due to the combined or synergistic effects of both volatile and non-volatile constituents present in the extract.

## 4. Materials and Methods

### 4.1. Identification and Collection of Haloxylon griffithii

A whole specimen of a *Haloxylon griffithii* plant was obtained in August 2021 in Quetta, Pakistan, and was identified and confirmed by Prof. Dr. Muhammad Zafar, Quaid-e-Azam University, Islamabad. A voucher specimen (215123) was deposited at the Pakistan Herbarium at Quaid-e-Azam University, Islamabad. Identification of the plant was alternatively validated through http://www.theplantlist.org, accessed on 31 August 2021.

### 4.2. Preparation of Crude Extract and Fractionations

The whole plant of *Haloxylon griffithii* was shade-dried for 10–12 days, crushed into a coarse powder with a mortar and pestle, and put through a sieve with a 0.3 mm aperture size. For crude extraction, approximately 500 g of the powder was subjected to Soxhlet extraction (65–85 °C) using 80% ethanol as the solvent. The extraction was carried out for 6–8 h until the solvent in the siphon tube appeared colorless, indicating complete extraction. The ethanolic extract was then concentrated under reduced pressure using a rotary evaporator at 40 °C to remove the solvent and obtain the crude extract. The resulting extract was stored at 4 °C in airtight containers for further analysis. A fresh portion of the powdered plant material was subjected to Soxhlet extraction with increasing polarity with *n*-hexane, ethyl acetate, ethanol, and water. The *n*-hexane, ethyl acetate, ethanol, and aqueous extracts were concentrated under reduced pressure with a rotary evaporator (BUCHI r-200, Büchi Labortechnik AG, Flawil, Switzerland) at 37 ± 2 °C and then kept in a refrigerator at 4 °C for later use.

### 4.3. Drugs and Chemicals

Chemicals, reagents, and standards were obtained from commercial sources (in parentheses) and were used as received without further purification. The following chemicals and standards were purchased from the Sigma Chemical Company (St. Louis, MO, USA): NaCl, KH_2_PO_4_, C_6_H_12_O_6_, CaCl_2_, NaH_2_PO_4_, KCl, MgSO_4_, MgCl_2_, NaHCO_3_, EDTA, acetylcholine chloride, atropine sulfate, loperamide hydrochloride, verapamil hydrochloride, charcoal, starch, and gum acacia. Castor oil was purchased from the local market.

### 4.4. Animals

Balb/c mice of either sex (18–25 g), Sprague–Dawley (SD) rats (200–250 g), and locally bred healthy rabbits (1–1.5 kg) of either sex were used throughout this investigation. These animals were kept in 12 h light/dark cycle at ambient room temperature and given free access to food and water at the Animal House of the Department of Pharmacy, Bahauddin Zakariya University, Multan, Pakistan. The animals were kept under standard conditions and rules of the Institute of Laboratory Animal Resources, Commission on Life Sciences, National Research Council [41] after the approval of the Ethical Committee of the Department of Pharmacy, Bahuaddin Zakariya University, Multan, Pakistan.

### 4.5. GC-MS Analysis

For GC-MS profiling, the crude hydroethanolic extract of *Haloxylon griffithii* was subjected to 1% dilution (w/v) with HPLC-grade methanol. The resulting extract was filtered with a 0.4-micron syringe filter and subjected to GC-MS analysis without further fractionation. GC-MS analysis of *H. griffithii* extracts was performed using the Agilent 7890B gas chromatograph (GC) (Agilent Technologies Inc., Santa Clara, CA, USA) coupled with mass selective detector 5977A (MSD), equipped with a DB5 (30 m × 250 µm × 0.25 µm, Agilent J&W, Santa Clara, CA, USA) capillary standard non-polar column. The oven temperature was set at 40 °C for 1 min, then increased to 10 °C per minute until it reached 260 °C, which was maintained for 28 min. The carrier gas (helium) flow rate was 1.2 mL/min, and the injection port temperature was 250 °C. The mass spectral (MS) source and quad temperatures were 230 °C and 150 °C, respectively. A split mode ratio of 10:1 was used for injecting the samples. The 40 to 650 (*m/z*) MS scan range was chosen for analysis [42]. Compounds were identified by comparing their retention times and mass spectra with those of authentic reference compounds stored in the DEMO.L and NIST20.L Library MS database and tabulated based on the CAS number (https://webbook.nist.gov/, analyzed 27 July 2024).

### 4.6. In-Silico Models

#### ADME Profiling of GC-MS-Identified Compounds

Selective bioactive compounds found from GC-MS analysis of *H. griffithii* were evaluated for absorption, distribution, metabolism, excretion, and toxicity (ADMET) in the “SwissADME” (http://www.swissadme.ch/, accessed on 28 January 2025) [43] and “PkCSM” (http://biosig.unimelb.edu.au/pkcsm/prediction, accessed on 28 January 2025) [44] to evaluate the ADMET and drug-likeness parameters. The ADMET properties, like intestinal absorption, volume of distribution, total renal clearance, ability to inhibit the P-glycoprotein, and hepatotoxicity, were also calculated using “PkCSM”.

### 4.7. In Vitro Investigation

#### Effect on Isolated Rabbit Jejunum

Experiments on the rabbit jejunum were carried out according to published procedures [22,24]. The animals were sacrificed and dissected to open the abdomen. After dissecting their abdomens, the jejunum tissues were carefully removed from the mesentery. Approximately 2 to 3 cm-long sections were cut and kept in freshly prepared Tyrode’s solution, which was aerated with carbogen gas (95% oxygen–5% carbon dioxide mixture) to keep them alive and ready for use. Each jejunum preparation was fixed in a 15 mL tissue organ bath maintained at 37 ± 0.5 °C filled with Tyrode’s solution and aerated with carbogen gas. Before test drug administration, a preload tensile strength of 1 ± 0.1 g was applied to each jejunal tissue preparation. Spontaneous recurrent contractions were observed after the jejunal tissue preparation was equilibrated for 15 ± 5 min by replacing Tyrode’s solution every 10 min with fresh Tyrode solution. For tissue response, we used an isotonic transducer (MLT0015, ADInstruments, Sydney, NSW, Australia) in conjunction with a data-accumulating system, Power Lab^®®^ (4/25), and recorded in Lab Chart Pro (Version 8, ADInstruments). The response was calculated based on the percentage of the contraction taken just before adding the test substance to determine whether the extracts exhibited spasmogenic or spasmolytic activity [45].

The antispasmodic effect of the *H. griffithii* extracts was measured by inducing smooth muscle contractions by a calcium ion channel blockade with high K^+^ (80 mM KCl). The extracts were then added cumulatively to get a dose-dependent inhibitory response. High K^+^ (80 mM KCl) causes an influx of calcium ions into the cell, thus changing the cell’s polarity and producing persistent contractions in isolated tissue. The inhibitory effect of the extract on this induced contraction was calculated and designated as the calcium channel-blocking activity of the extract. Concentration-based response curves (CRCs) of calcium were constructed in the presence and absence of the extracts to further confirm the mechanism of action. To establish these curves, first, jejunal tissues were thrice incubated with K^+^ (80 mM) and then stabilized in EDTA-containing calcium-free Tyrode solution for about 30 ± 5 min, followed by 50 ± 5 min incubation in potassium-rich and calcium-free Tyrode solution, to eventually diminish the intracellular calcium stores. After the equilibration of tissues, calcium was then added to the tissue organ bath cumulatively to construct control CRCs. After the control curves, the tissues were washed and incubated for 50±5 min with varying doses of extracts, the curves were reconstructed, and the results were compared with the control CRCs [19].

### 4.8. In Vivo Investigations

#### 4.8.1. Castor Oil-Induced Diarrhea Model

Blab/C mice (20–30 g) of either sex were randomly divided into seven groups of five mice each (*n* = 5). The animals were starved overnight (16 h) and given free access to water before the experiments. The following treatments were given orally:

Group 1: (Negative Control group) received saline solution (10 mL/kg);

Group 2: (Disease control group) received saline solution (10 mL/kg);

Group 3 (standard group) received 10 mg/kg of loperamide and was designated as the standard control group;

Group 4 (standard group) received verapamil 10 mg/kg and was designated as the standard control group 2;

Groups 5, 6, and 7 received various concentrations of Hg.Cr (100, 200, and 400 mg/kg, respectively).

Furthermore, the animals were given access to water before the administration of castor oil. One hour later, all groups except group 1 received castor oil via oral gavage at 10 mL/kg. Each animal was caged individually with flooring lined with blotting paper replaced every hour. The number of diarrheal stools was recorded over 4 h. The stools from the groups treated with Hg.Cr were compared to those of the positive control group, which received only castor oil. Data were reported as the number of defecations and their percentage representation [46]. The percentage protection from diarrhea was calculated according to the following equation:

Percent (%) protection = [(T1 − E)/ T1] × 100;

where T1 represents the average number of defecations induced by the administration of castor oil, and E signifies the average number of defecations caused by the administration of the extract.

#### 4.8.2. Charcoal Meal GIT Transit Time

This test was performed according to the procedure outlined by Wahid et al. (2022) [25], with slight modifications to assess gastrointestinal motility. In this test, rats were randomly assigned to groups (*n* = 5 per group), starved for 14 h before the experiment, and allowed free access to drinking water. The groups received one of the following treatments orally:

Group 1 (Control): 0.9% normal saline (10 mL/kg);

Group 2 (Standard Drug): loperamide (10 mg/kg);

Group 3 (Standard Drug): verapamil (10 mg/kg);

Groups 4 and 5 (Hg.Cr) Hg.Cr at 200 and 400 mg/kg, respectively;

Groups 6 and 7 (Hg.Hex) Hg.Hex at 200 and 400 mg/kg, respectively;

Groups 8 and 9 (Hg.EA) Hg.EA at 200 and 400 mg/kg, respectively;

Groups 10 and 11 (Hg.OH): Hg.OH at 200 and 400 mg/kg, respectively;

Group 12 (Hg.Aq): Hg.Aq at 200 and 400 mg/kg, respectively.

Fifteen minutes after administering the treatment, each rat received a 10 mL/kg charcoal meal suspension. This suspension was made using distilled water and contained 10% gum acacia, 20% starch, and 10% charcoal. After thirty minutes of charcoal meal administration, the rats were euthanized by cervical dislocation, and their small intestines were removed after dissecting their abdomens. The distance the charcoal meal traveled was measured, starting 3 cm from the pylorus to the furthest point reached by the charcoal. The results were reported as the charcoal meal transport ratio, representing the percentage of gastrointestinal motility.

The following equation was used to calculate the percentage of gastrointestinal motility:

Charcoal Meal Transport Ratio (%) = [D1/D2] × 100;

where D1 = distance covered by charcoal and D2 = total intestinal length

### 4.9. Statistical Analysis

All determinations were conducted in triplicate, and the data were subjected to a one-way analysis of variance (ANOVA) and a two-way ANOVA, which were additionally analyzed using Dunnett’s test. The results are expressed as the mean ± standard error of the mean (S.E.M). The half maximal effective concentration (EC_50_) values are given as a geometric mean with 95% confidence intervals (CI). Statistical analysis was performed using GraphPad Prism 8.0.1. (GraphPad Software, San Diego, CA, USA, http://www.graphpad.com); the differences were significant at *p* ≤ 0.05 [47].

## 5. Conclusions

The present study contributes to the existing knowledge on *Haloxylon griffithii* by providing preliminary experimental support for its traditional use in gastrointestinal disorders. While the findings are promising, further in-depth phytochemical and mechanistic investigations are warranted to fully validate its therapeutic potential. A comprehensive pharmacological investigation demonstrated the plant’s significant antispasmodic and antidiarrheal properties, particularly in non-polar fractions. These effects were mechanistically linked to calcium channel-blocking activity, supported by both in vitro jejunum assays and calcium response studies. The lead molecules, including fatty acid esters, monoterpenes, and a rare azulene derivative, exhibit therapeutic effects that can be utilized as natural alternatives to synthetic drugs with fewer side effects. Even though such results provide promising prospects, more studies must be carried out to isolate individual bioactive constituents, ascertain their mechanisms of action, and verify their efficacy in a clinical setup.

## Figures and Tables

**Table 1:** Chief components identified in *Haloxylon griffithii* by GC-MS analysis.

Sr. No.	Peak no.	RT	% Area	Compound Name	Mol. Weight g/mol	Mol. Formula
1	2	7.703	0.10	*o*-Cymene	134.2182	C_10_H_14_
2	4	11.750	0.18	Cyclohexasiloxane, dodecamethyl-	444.9236	C_12_H_36_O_6_Si_6_
3	7	13.958	0.41	Cycloheptasiloxane, tetradecamethyl-	519.0776	C_14_H_42_O_7_Si_7_
4	9	14.29	0.15	Pentacosane	352.6804	C_25_H_52_
5	11	14.541	0.04	2,4-Di-*tert*-butylphenol	206.3239	C_14_H_22_O
6	12	15.930	0.25	Cyclooctasiloxane, hexadecamethyl-	593.2315	C_16_H_48_O_8_Si_8_
7	15	16.499	0.11	2-Propenal, 3-(2,6,6-trimethyl-1-cyclohexen-1-yl)-	178.2707	C_12_H_18_O
8	16	16.808	0.17	2-Hexen-1-ol, 2-ethyl	128.2120	C_8_H_16_O
9	19	17.631	0.11	Cyclononasiloxane, octadecamethyl	667.3855	C_18_H_54_O_9_Si_9_
10	23	19.045	0.15	7,9-Di-*tert*-butyl-1-oxaspiro(4,5)deca-6,9-diene-2,8-dione	276.3707	C_17_H_24_O_3_
11	24	19.146	0.18	Cyclodecasiloxane, eicosamethyl-	741.5394	C_20_H_60_O_10_Si_10_
12	25	19.197	0.13	Hexadecanoic acid, methyl ester	270.4507	C_17_H_34_O_2_
13	27	19.533	0.34	*n*-Hexadecanoic acid	256.4241	C_16_H_32_O_2_
14	28	19.873	0.48	Hexadecanoic acid, ethyl ester	284.4772	C_18_H_36_O_2_
15	29	20.019	0.13	(3aR,4R,7R)-1,4,9,9-Tetramethyl-3,4,5,6,7,8-hexahydro-2*H*-3a,7-methanoazulen-2-one	218.3346	C_15_H_22_O
16	32	20.542	0.82	Benzene, 1,2,4,5-tetrakis(1-methylethyl)-	246.4308	C_18_H_30_
17	35	20.838	0.11	9,12-Octadecadienoic acid (Z,Z)-, methyl ester	294.48	C_19_H_34_O_2_
18	36	20.929	0.44	Benzene, hexaethyl-	246.4308	C_18_H_30_
19	38	21.182	0.36	9,12-Octadecadienoic acid (Z,Z)-	280.4455	C_18_H_32_O_2_
20	40	21.460	0.90	Linoleic acid ethyl ester	308.4986	C_20_H_36_O_2_
21	41	21.515	0.67	(*E*)-9-Octadecenoic acid ethyl ester	310.5145	C20H38O2
22	44	22.024	33.57	α-Santonin	246.3016	C_15_H_18_O_3_
23	58	24.963	43.93	Bis(2-ethylhexyl) phthalate	390.5561	C_24_H_38_O_4_

**Table 2:** Comparative ADME profiling of compounds identified from GC-MS analysis of *H. griffithii* crude extract and Verapamil.

	Molecule	Verapamil	Alpha Santonin	O-Cymene	Hexadecanoic Acid, Ethyl Ester	Hexadecanoic Acid, Methyl Ester	*n*-Hexadecanoic Acid	(E)-9-Octadecenoic Acid Ethyl Ester	Bis(2-ethylhexyl) Phthalate	Benzene, Hexaethyl-	Cyclodecasiloxane, Eicosamethyl-
Basic properties	Formula	C_27_H_38_N_2_O_4_	C_15_H_18_O_3_	C_10_H_14_	C_18_H_36_O_2_	C_17_H_34_O_2_	C_16_H_32_O_2_	C_20_H_38_O_2_	C_24_H_38_O_4_	C_18_H_30_	C_20_H_60_O_10_Si_10_
	MW (g/mol)	454.6	246.3	134.22	284.48	270.45	256.42	310.51	390.56	246.43	741.54
	iLOGP	4.5	2.25	2.43	4.65	4.41	3.85	5.03	4.77	3.85	6.55
	XLOGP3	3.79	2.29	4.38	7.88	7.38	7.17	8.03	7.45	6.7	10.06
	WLOGP	5.09	2.42	3.12	6.03	5.64	5.55	6.59	6.43	5.06	7.18
	MLOGP	2.96	2.38	4.47	4.67	4.44	4.19	5.03	5.24	6.57	–2.43
Absorption	GI absorption	High	High	Low	High	High	High	Low	High	Low	Low
	Pgp substrate	Yes	No	No	No	No	No	No	Yes	No	Yes
	Bioavailability Score	0.55	0.55	0.55	0.55	0.55	0.85	0.55	0.55	0.55	0.55
	Skin Permeability log Kp (cm/s)	–6.38	–6.18	–4.01	–2.44	–2.71	–2.77	–2.49	–3.39	–3.05	–3.68
Distribution	BBB permeant	Yes	Yes	Yes	No	Yes	Yes	No	No	No	No
	BBB permeability log BB	–0.647	0.347	0.48	0.759	0.749	–0.111	0.786	–0.175	0.741	–2.905
	VDss (human) log L/kg	0.931	0.205	0.744	0.373	0.334	–0.543	0.332	0.36	1.334	–0.106
Metabolism	CYP1A2 inhibitor	No	No	No	Yes	Yes	Yes	Yes	No	No	No
	CYP2C19 inhibitor	No	No	No	No	No	No	No	No	No	No
	CYP2C9 inhibitor	No	No	No	No	No	Yes	No	Yes	No	No
	CYP2D6 inhibitor	Yes	No	Yes	No	No	No	No	No	No	No
	CYP3A4 inhibitor	Yes	No	No	No	No	No	No	Yes	No	No
Excretion	Total Clearance logml/min/kg	1.072	0.197	0.259	1.912	1.861	1.763	2.03	1.898	2.03	−0.484
Toxicity	Hepatotoxicity	No	No	No	No	No	No	No	No	Yes	No
	Skin Sensitisation	No	No	Yes	Yes	Yes	Yes	yes	No	yes	No
Drug Likeness	Lipinski #violations	0	0	1	1	1	1	1	1	1	1
	Ghose #violations	2	0	1	1	1	0	1	1	0	4
	Veber #violations	1	0	0	1	1	1	1	1	0	0
	Egan #violations	0	0	0	1	0	0	1	1	0	1
	Muegge #violations	0	0	2	2	1	1	2	2	2	2

The physicochemical and pharmacokinetic properties listed in this table were primarily obtained through computational prediction using online tools such as SwissADME (http://www.swissadme.ch, accessed on 28 January 2025) and pkCSM (http://biosig.unimelb.edu.au/pkcsm, accessed on 28 January 2025). Compound identification and basic molecular data (e.g., molecular formula, retention time) were confirmed using the NIST Chemistry WebBook and PubChem.

**Table 3:** Effect of the hydroethanolic extract of Hg.Cr on castor oil-induced diarrhea in mice (*n* = 5).

Groups	Dose		No. of Wet Feces			
		0–0.5 h	0–1 h	0–2 h	0–3 h	0–4 h
Group I (Negative control)	10 mL/kg (NS)	0.2	1.0	1.0	2.0	4.0
Group II (Disease control)	10 mL/kg (NS)	5.0	11.0	18.0	29.0	32.0
Group III (standard)	10 mg/kg (Loperamide)	0.4	2.0	3.0	6.0	7.0
Group IV (standard)	10 mg/kg (verapamil)	2.0	3.0	4.0	4.8	5.0
Group V (Treatment)	100 mg/kg (Hg.Cr)	5.4	11.0	18.0	19.6	21.0
0Group VI (Treatment)	200 mg/kg (Hg.Cr)	5.2	9.0	13.0	17.0	19.8
Group VII (Treatment)	400 mg/kg (Hg.Cr)	3.0	7.0	8.0	8.6	10.0

**Table 4:** Percentage protection against castor oil-induced diarrhea by hydroethanolic plant extract (Hg.Cr) (*n* = 5; data are presented as the mean ± SEM).

Groups	Dose	Total Number of Feces	%Age Protection
Group I (Negative control)	10 mL/kg (NS)	4.0 ± 0.45	87.5
Group II (Disease control)	10 mL/kg (NS)	32.0 ± 1.05	00.0
Group III (standard)	10 mg/kg (Loperamide)	7.0 ± 0.71	78.1
Group IV (standard)	10 mg/kg (Verapamil)	5.0 ± 0.45	84.4
Group V (Treatment)	100 mg/kg (Hg.Cr)	21.0 ± 0.63	34.4
Group VI (Treatment)	200 mg/kg (Hg.Cr)	19.8 ± 0.86	38.1
Group VII (Treatment)	400 mg/kg (Hg.Cr)	10.0 ± 0.95	68.7

## Data Availability

Data is contained within the article.

## Abbreviations

The following abbreviations are used in this manuscript:

*H. griffithii*	*Haloxylon griffithii*
CRCs	Concentration response curves
Hg.Cr	*Haloxylon griffithii* crude extract
Hg.Hex	*Haloxylon griffithii* n-hexane fraction
Hg.EA	*Haloxylon griffithii* ethyl acetate fraction
Hg.OH	*Haloxylon griffithii* ethanolic fraction
Hg.Aq	*Haloxylon griffithii* Aqueous fraction
GIT	Gastrointestinal tract
VGCC	Voltage-gated calcium channels

## Appendix A

**Table A1:** List of all volatile and semi-volatile compounds identified in the crude hydroethanolic extract of *Haloxylon griffithii* using GC-MS analysis.

Peak no.	RT	% Area	Compound Name	Hit Quality
1	3.466	0.21	2-Butanone, 4-hydroxy-3-methyl-	52
2	7.703	0.10	*o*-Cymene	95
3	10.480	0.14	4-Hydroxy-.beta.-thujone	72
4	11.750	0.18	Cyclohexasiloxane, dodecamethyl-	93
5	13.215	0.12	1-(1-Butyny)cyclopentanol	50
6	13.677	0.26	Hexanoic acid, 3,7-dimethyl-6-octenyl ester	49
7	13.958	0.41	Cycloheptasiloxane, tetradecamethyl-	93
8	14.126	0.15	Carane, 4,5-epoxy-, trans	46
9	14.29	0.15	Pentacosane	74
10	14.38	0.06	Ledol	38
11	14.541	0.04	2,4-Di-*tert*-butylphenol	78
12	15.930	0.25	Cyclooctasiloxane, hexadecamethyl-	94
13	16.174	0.03	Bicyclo [2.2.1]heptane, 1,3,3-trimethyl	46
14	16.217	0.04	2-Heptenoic acid, methyl ester, (E)-	44
15	16.499	0.11	2-Propenal, 3-(2,6,6-trimethyl-1-cyclohexen-1-yl)-	59
16	16.808	0.17	2-Hexen-1-ol, 2-ethyl	89
17	16.979	0.23	(S,E)-6-Hydroxy-6-methyl-2-((2S,5R)-5-methyl-5-vinyltetrahydrofuran-2-yl)	68
18	17.315	0.14	1H-Indene, 1-ethylideneoctahydro-7a-methyl-, (1Z,3a.alpha.,7a.beta.)	87
19	17.631	0.11	Cyclononasiloxane, octadecamethyl	90
20	18.154	0.12	Isopropyl myristate	35
21	18.536	0.14	7,10,10-Trimethyl-4-oxa-3,5-diazatricyclo [5.2.1.0(2,6)]deca-2,5-dien-3-one	35
22	18.959	0.14	2,6-Pyrazinedicarbonitrile, 3-amino-5-(diethylamino)-	41
23	19.045	0.15	7,9-Di-*tert*-butyl-1-oxaspiro(4,5)deca-6,9-diene-2,8-dione	99
24	19.146	0.18	Cyclodecasiloxane, eicosamethyl-	52
25	19.197	0.13	Hexadecanoic acid, methyl ester	96
26	19.419	0.10	5-(6-Methyl-7-oxooctyl)-2(5H)-furanone (isomer 2)	47
27	19.533	0.34	*n*-Hexadecanoic acid	96
28	19.873	0.48	Hexadecanoic acid, ethyl ester	99
29	20.019	0.13	(3aR,4R,7R)-1,4,9,9-Tetramethyl-3,4,5,6,7,8-hexahydro-2*H*-3a,7-methanoazulen-2-one	78
30	20.216	1.89	Lumisantonin	95
31	20.326	0.92	Propionic acid, 3-(2-methyl-4-oxo-2,3,4,5-tetrahydrobenzo[b][1,4]diazepin-1-yl)-	44
32	20.542	0.82	Benzene, 1,2,4,5-tetrakis(1-methylethyl)-	89
33	20.651	0.12	6-Hydroxy-5a,9-dimethyl-3-methylidene-4,5,6,7,9a,9b-hexahydro-3aH-benzo[g][1]benzofuran-2-one	66
34	20.760	0.20	2-Butanone, 4-(2,6,6-trimethyl-2-cyclohexen-1-ylidene)-	80
35	20.838	0.11	9,12-Octadecadienoic acid (Z,Z)-, methyl ester	91
36	20.929	0.44	Benzene, hexaethyl-	89
37	20.990	0.33	Cyclohexanol, 5-methyl-2-(1-methylethyl)-, (1.alpha.,2.alpha.,5.alpha	43
38	21.182	0.36	9,12-Octadecadienoic acid (Z,Z)-	95
39	21.235	1.55	4(1H)-Quinolinone,7-amino-3-carboxy-6-fluoro-1-ethyl	44
40	21.460	0.90	Linoleic acid ethyl ester	99
41	21.515	0.67	(*E*)-9-Octadecenoic acid ethyl ester	95
42	21.707	0.18	3,6-Nonadien-5-one, 2,2,8,8-tetramethyl	46
43	21.778	0.25	3-Isopropoxy-1,1,1,7,7,7-hexamethyl-3,5,5-tris(trimethylsiloxy)tetrasiloxane	27
44	22.024	33.57	α-Santonin	99
45	22.123	1.22	N-[1-Ethylpyridinylidene]-1,3,2-dioxaphospholan-2-amine 2-sulfide	42
46	22.260	3.11	Phenanthrene, 3,6-dimethoxy-9,10-dimethyl	59
47	22.305	2.30	Alantolactone, 4.alpha.,4A.alpha.-epoxy	64
48	22.615	0.30	(-)-Neoclovene-(II), dihydro	90
49	22.668	0.34	Verrucarol	62
50	22.941	0.31	Oxazepam, 2TMS derivative	56
51	23.039	0.23	2H-3,9a-Methano-1-benzoxepin, octahydro-2,2,5a,9-tetramethyl-, [3R-(3.alpha.,5a.alpha.,9.alpha.,9a.alpha.)]-	56
52	23.203	0.13	Spiro[2.5]octane, 5,5-dimethyl-4-(3-oxobutyl)-	30
53	23.351	0.17	Ethanone, 1-(1,2,3,4,7,7a-hexahydro-1,4,4,5-tetramethyl-1,3a-ethano-3aH-inden-6-yl)-	47
54	23.545	0.26	Benzothiophene-3-carboxylic acid, 4,5,6,7-tetrahydro-2-amino-6-ethyl-, ethyl ester	38
55	23.850	0.11	2-Pyridinamine, N-(4,5-dihydro-5-methyl-2-thiazolyl)-3-methyl	47
56	24.383	0.19	Bis(2-ethylhexyl) phthalate	68
57	24.563	0.19	2-N,2-N,4-N,4-N,8-Pentamethylquinoline-2,4,5-triamine	70
58	24.963	43.93	Bis(2-ethylhexyl) phthalate	90
59	27.919	0.09	1,4-Benzenedicarboxylic acid, bis(2-ethylhexyl) ester	94

**Table A2:** 2D-Structures of chief components identified in *Haloxylon griffithii* by GCMS analysis.

S. No.	Peak no.	Compound Name	Mol. Weightg/mol	Mol. Formula	Structure
1	2	*o*-Cymene	134.2182	C_10_H_14_	
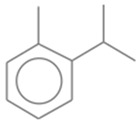
2	4	Cyclohexasiloxane, dodecamethyl-	444.9236	C_12_H_36_O_6_Si_6_	
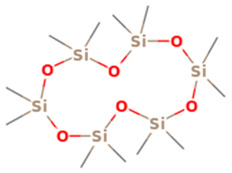
3	7	Cycloheptasiloxane, tetradecamethyl-	519.0776	C_14_H_42_O_7_Si_7_	
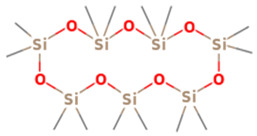
4	9	Pentacosane	352.6804	C_25_H_52_	
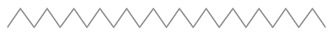
5	11	2,4-Di-*tert*-butylphenol	206.3239	C_14_H_22_O	
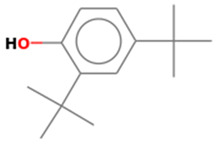
6	12	Cyclooctasiloxane, hexadecamethyl-	593.2315	C_16_H_48_O_8_Si_8_	
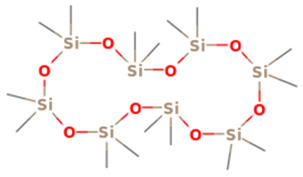
7	15	2-Propenal, 3-(2,6,6-trimethyl-1-cyclohexen-1-yl)-	178.2707	C_12_H_18_O	
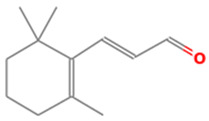
8	16	2-Hexen-1-ol, 2-ethyl	128.2120	C_8_H_16_O	
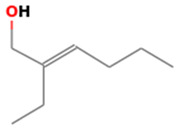
9	19	Cyclononasiloxane, octadecamethyl	667.3855	C_18_H_54_O_9_Si_9_	
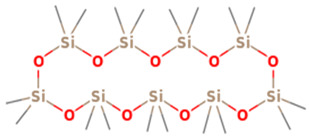
10	23	7,9-Di-*tert*-butyl-1-oxaspiro(4,5)deca-6,9-diene-2,8-dione	276.3707	C_17_H_24_O_3_	
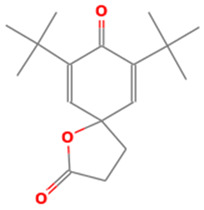
11	24	Cyclodecasiloxane, eicosamethyl-	741.5394	C_20_H_60_O_10_Si_10_	
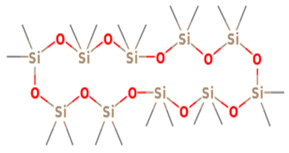
12	25	Hexadecanoic acid, methyl ester	270.4507	C_17_H_34_O_2_	
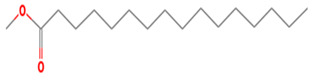
13	27	*n*-Hexadecanoic acid	256.4241	C_16_H_32_O_2_	
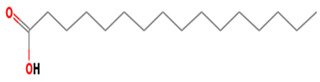
14	28	Hexadecanoic acid, ethyl ester	284.4772	C_18_H_36_O_2_	
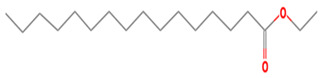
15	29	(3aR,4R,7R)-1,4,9,9-Tetramethyl-3,4,5,6,7,8-hexahydro-2*H*-3a,7-methanoazulen-2-one	218.3346	C_15_H_22_O	
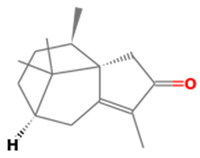
16	32	Benzene, 1,2,4,5-tetrakis(1-methylethyl)-	246.4308	C_18_H_30_	
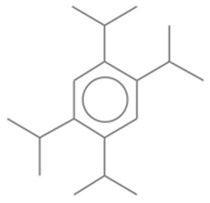
17	35	9,12-Octadecadienoic acid (Z,Z)-, methyl ester	294.48	C_19_H_34_O_2_	
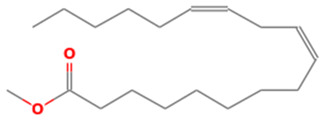
18	36	Benzene, hexaethyl-	246.4308	C_18_H_30_	
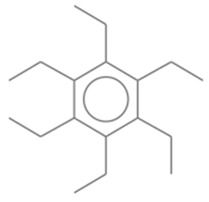
19	38	9,12-Octadecadienoic acid (Z,Z)-	280.4455	C_18_H_32_O_2_	
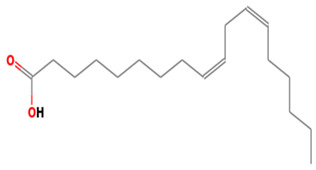
20	40	Linoleic acid ethyl ester	308.4986	C_20_H_36_O_2_	
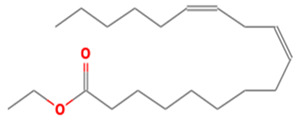
21	41	(*E*)-9-Octadecenoic acid ethyl ester	310.5145	C20H38O2	
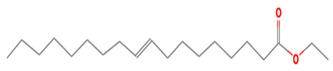
22	44	α-Santonin	246.3016	C_15_H_18_O_3_	
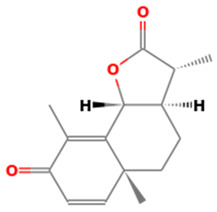
23	58	Bis(2-ethylhexyl) phthalate	390.5561	C_24_H_38_O_4_	
